# ChopSticks: High-resolution analysis of homozygous deletions by exploiting concordant read pairs

**DOI:** 10.1186/1471-2105-13-279

**Published:** 2012-10-30

**Authors:** Tomohiro Yasuda, Shin Suzuki, Masao Nagasaki, Satoru Miyano

**Affiliations:** 1 Human Genome Center, Institute of Medical Science, University of Tokyo, 4-6-1 Shirokanedai, Minato-ku, Tokyo 108-8639, Japan; 2 Department of Integrative Genomics, Tohoku Medical Megabank Organization, Tohoku University, 2-1 Seiryo-machi, Aoba-ku, Sendai 980-8573, Japan

## Abstract

**Background:**

Structural variations (SVs) in genomes are commonly observed even in healthy individuals and play key roles in biological functions. To understand their functional impact or to infer molecular mechanisms of SVs, they have to be characterized with the maximum resolution. However, high-resolution analysis is a difficult task because it requires investigation of the complex structures involved in an enormous number of alignments of next-generation sequencing (NGS) reads and genome sequences that contain errors.

**Results:**

We propose a new method called *ChopSticks* that improves the resolution of SV detection for homozygous deletions even when the depth of coverage is low. Conventional methods based on read pairs use only *discordant* pairs to localize the positions of deletions, where a discordant pair is a read pair whose alignment has an aberrant strand or distance. In contrast, our method exploits concordant reads as well. We theoretically proved that when the depth of coverage approaches zero or infinity, the expected resolution of our method is asymptotically equal to that of methods based only on discordant pairs under double coverage. To confirm the effectiveness of ChopSticks, we conducted computational experiments against both simulated NGS reads and real NGS sequences. The resolution of deletion calls by other methods was significantly improved, thus demonstrating the usefulness of ChopSticks.

**Conclusions:**

ChopSticks can generate high-resolution deletion calls of homozygous deletions using information independent of other methods, and it is therefore useful to examine the functional impact of SVs or to infer SV generation mechanisms.

## Background

Today, next-generation sequencing (NGS) technologies are essential tools in genome analysis, because they enable us to simultaneously obtain sequences of up to hundreds of billions of base pairs [1]. These technologies enable the characterization of not only small variations such as single-nucleotide polymorphisms (SNPs) but also large-scale mutations such as insertions, deletions, tandem duplications, and inversions. Mutations of these types are collectively called structural variations (SVs) and are frequently observed even in healthy individuals [2-4]. Because SVs affect a much larger portion of genomes than small variations, including SNPs, they have a great impact on biological functions.

Current NGS methods can sequence paired reads, which are pairs of reads several hundred bases away from each other. This ability is useful for analyzing SVs because paired reads can be aligned with the reference genome more accurately than single reads, and because we can analyze structures of genomes larger than the size of each read. However, SV detection is still a difficult task, because it requires analysis of the complex structures involved in an enormous number of alignments of paired reads with the reference genome, and because read sequences and alignments include unavoidable errors. Therefore, for example, a false detection rate (FDR) up to 10% had to be tolerated even when determining just the existence of each SV in the 1000 Genomes Project [2]. It is obviously more difficult to accurately detect the exact positions of SVs. Nevertheless, high-resolution SV calls are necessary to elucidate the functional impact of SVs and molecular mechanisms that generate SVs. Moreover, to conduct a large-scale analysis, SV detection methods for data with a low depth of coverage (hereafter simply referred to as *coverage*) are desirable, because whole genome sequencing is not easy even with NGS technologies.

Current methods for SV detection search for *signatures* that indicate SVs hidden in read sequences and their alignments with the genome sequences. The following are basic signatures used for SV detection [2-4]. 

• Read pair (RP) [5-7]: If pairs of reads have aberrant strands or distances, they are likely to be caused by SVs. Such pairs are called *discordant* pairs, and normally mapped ones are called *concordant* pairs. If strands of a discordant pair are as expected, a larger distance than expected indicates a deletion, whereas a smaller distance indicates an insertion. There are several categories of methods that detect discordant pairs by using mapping distances. 

• Threshold-based: A pair with a mapped distance larger or smaller than a predefined threshold is defined as a discordant pair. The threshold is *μ*±3 *σ*or *μ*±4 *σ*for BreakDancer [5] and VariationHunter [6] where *μ*and *σ*are mean and standard deviation of mapping distances, or median fragment size ± 10 median absolute deviations for HYDRA [7].

• Distribution-based: Although the mapped distance of a single pair might vary by tens or hundreds bases even without SVs, larger (smaller) mapping distances of many pairs in the same region indicate deletions (insertions). Such reads can be detected by statistical tests on the distribution of mapped distances [5,8]. Pairs detected in this way might have mapping distances more similar to the expected distance than those of other methods. Nonetheless, we still call them *discordant* pairs in this paper to unify the word used to refer pairs that support SVs.

• Graph-based: Recently Marshall et al. [9] proposed a new method CLEVER based on the graph theory. CLEVER constructs a graph where a node represents an alignment of a read pair and the genome, while an edge means that connected alignments potentially support the same allele. In this graph, a clique corresponds to a set of pairs supporting the same allele. CLEVER detects SVs by finding maximal cliques (max-cliques). CLEVER has an ability to find more than one max-clique overlaping each other, each of which supports a different allele. Therefore CLEVER can distinguish more than one SV located at the same locus, for example, two deletions of different sizes in a diploid genome.

• Read depth (RD) [10,11]: If coverage changes at some position in the genome, this indicates a copy number variation.

• Split read (SR) [12]: If an alignment of a read and the genome includes only a part of the read, this indicates a position of a breakpoint. Here, a *breakpoint* is the boundary between a region affected by some SV and its unaffected flanking region.

• Sequence assembly (AS) [7,13]: If the coverage is sufficient, assembling NGS reads around an SV reveals the exact sequence around the SV and the positions of breakpoints.

The most popular signature used to detect SVs is threshold-based RP. Methods based on this signature can detect SVs from a small number of discordant read pairs; therefore threshold-based RP methods can be applied to low-coverage data. However, threshold-based RP methods localize SVs only to regions surrounded by discordant read pairs, thus causing some ambiguity. For RD methods, the problem of resolution is much bigger. Because RD methods involve calculation of coverage in windows of a fixed size, its resolution cannot be finer than the window size. Methods based on the SR signature can determine positions of breakpoints up to base-pair-level (bp-level) resolution if there are reads covering the breakpoints. However, such reads might not exist, in particular when coverage is low, because of unevenness of coverage or repeat elements to which reads cannot be aligned uniquely. Moreover, because such a split alignment is shorter than a read itself, careful analysis is required to avoid spurious matches. If coverage is sufficiently high, AS methods would ultimately reveal the exact positions of SVs at bp-level resolution. Although extremely deep sequencing can be conducted by targeted sequencing [14], it is still expensive to obtain paired reads of high coverage over the entire genome so that assembly can be performed. In fact, a previous study has indicated that the sensitivity of AS methods is rather low (Table S6B of Mills et al. [3]).

Because these signatures have their own advantages and disadvantages, it is desirable to combine more than one method [4]. In fact, several methods that use more than one signature have been proposed recently [15,16]. In combined approaches, we should integrate SV signatures that are independent of each other. In this paper, we propose a new method called *ChopSticks* that improves the resolution of deletion calls for homozygous deletions generated mainly by threshold-based RP methods. ChopSticks is especially valuable when target SVs are expected to be homozygous as those of inbred mice whose genomes are homozygous at virtually all loci [17]. ChopSticks exploits positions of concordant read pairs in addition to those of discordant ones. Thus far, they have been ignored in threshold-based RP approaches, and therefore, our method can improve the resolution by using this new independent information. As explained below, ChopSticks is effective even for data whose coverage is low.

The organization of this paper is as follows. First, we theoretically analyze the improvement of the resolution achieved by exploiting concordant read pairs. Next, we present our computational method ChopSticks that improves the resolution of homozygous deletion calls. After that, we demonstrate the effectiveness of ChopSticks in computational experiments. Then, we present our conclusions. In addition, we illustrate details of our method and experiments in Methods section.

## Results and discussion

### Strategy for resolution improvement

#### Theoretical estimation of resolution

Here we present results of our theoretical analysis of improved resolution achieved by our method as compared to RP methods. We also present the necessary definitions to describe them. See Methods for details.

We define a *discordant read* as a read of a discordant pair and a *concordant read* as that of a concordant pair. Among the two reads of a pair, the one mapped upstream is called an *upstream read* and the other is called a *downstream read* in this paper. Let *c* be the depth of coverage. Assume that the positions of read pairs are uniformly random over the genome, and that the length *r* of each read is a fixed constant. Let *q*(*c*) be the probability that there is no read pair whose upstream read begins at a given base in the genome. Suppose that there are *N* read pairs uniquely mapped to a genomic sequence of length *G*. According to a classical analysis [18],

(1) $$\begin{array}{l} q(c)={\left(1-\frac{1}{G}\right)}^{N}\approx e^{-N/G}=e^{-c/2r}. \end{array}$$

Hereafter, we just write *q* instead of *q*(*c*) for simplicity. In threshold-based RP approaches, the predicted position of an upstream end of a deletion is determined by the upstream discordant read that is the closest to the breakpoint. Let *b* be the position of an upstream end of a deletion, *Δ*_*b*_ be the distance between *b* and the closest upstream discordant read, and *d* be the distance between paired reads. We assume that *d* is a constant. Let *E*[*Δ*_*b*_|*b*,*c*] be the expectation of *Δ*_*b*_given that *b* is detected and the coverage is *c*. Then,

(2) $$\begin{array}{l} E[\Delta_{b}|b,c]=\frac{1-q}{1-q^{d+1}}S(q,d), \end{array}$$

where

$$S(q,d)=\overset{d}{\underset{j=0}{\sum }}jq^{j}=\frac{q-(d+1)q^{d+1}+dq^{d+2}}{{\left(1-q\right)}^{2}}.$$

**Figure 1:**
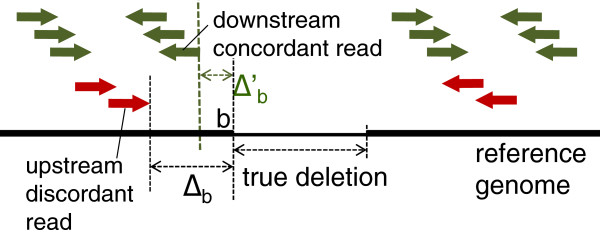
**Resolution improvement by exploiting concordant read pairs.** Schematic illustration of the key idea of our method ChopSticks. Unlike conventional SV detection methods based only on discordant pairs whose mapping distances were not close to the expectation, ChopSticks uses concordant read pairs as well. There is a chance that there is a concordant read closer to the boundary of the deleted region (breakpoint) than any discordant reads. Such a concordant read localizes the predicted position of the breakpoint, and therefore it contributes to achieving a high resolution. In this figure, *b* is the upstream end of a true deletion, *Δ*_*b *_is the distance between the upstream end of a true deletion and that of a deletion call by threshold-based read-pair (RP) methods. Similarly, $\Delta_{b}^{\prime }$ is defined for our method. The expected values of *Δ*_*b*_and $\Delta_{b}^{\prime }$ are given by Equations (2) and (3), respectively.

See Methods for derivation of Equation (2). We can obtain better resolution by using concordant reads in addition to discordant reads, because there is a chance that there exists a concordant read closer to *b* than any upstream discordant read (Figure 1). Such a read can contribute to the localization of the position where *b* can exist. Let $\Delta_{b}^{\prime }$ be the distance between *b* and the closest read in the upstream of *b*, and let $E[\Delta_{b}^{\prime }|b,c]$ be the expectation of $\Delta_{b}^{\prime }$ given that *b* is detected and the coverage is *c*. Then,

(3) $$\begin{array}{lll} \;E[\Delta_{b}^{\prime }|b,c]= & \frac{1}{1-q^{d+1}}\; & \;\\ & \times \left(\left(1-q^{2})S(q^{2},d)-q^{d+1}(1-q)S(q,d\right)\right).\; \end{array}$$

**Figure 2:**
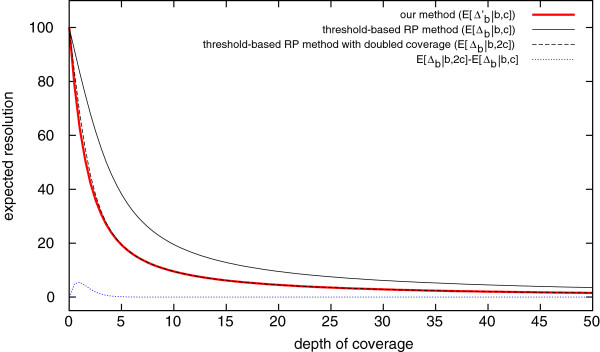
**Expected resolutions of ChopSticks and threshold-based RP methods.** The expected resolution of our method ($E[\Delta_{b}^{\prime }|b,c]$) is shown by a thick red line, that of threshold-based RP methods (*E*[*Δ*_*b*_|*b*,*c*]) is shown by a thin solid black line, and that of threshold-based RP methods with double coverage (*E*[*Δ*_*b*_|*b*,2*c*]) is shown by a dashed black line. The difference between $E[\Delta_{b}^{\prime }|b,c]$ and *E*[*Δ*_*b*_|*b*,2*c*] is also shown by a dotted blue line. As the coverage goes away from zero, the resolution obtained by our method quickly outperforms that of normal RP methods. It is also clear that the resolution of our method is very close to that of threshold-based RP methods with double coverage. The difference approaches zero when coverage approaches zero or infinity, as indicated by the blue dotted line. *E*[*Δ*_*b*_|*b*,*c*], $E[\Delta_{b}^{\prime }|b,c]$, and *E*[*Δ*_*b*_|*b*,2*c*] are given by Equations (2), (3), and (5), respectively. In this figure, *d*=200 and *r*=100.

As shown in Figure 2, the expected resolution of our method is significantly superior to that of threshold-based RP methods, which only use discordant pairs. The achieved resolution is quite close to that of threshold-based RP methods but with double coverage, which we confirmed theoretically.

##### Theorem 1

The expectation $E[\Delta_{b}^{\prime }|b,c]$ is a weighted sum of *E*[*Δ*_*b*_|*b*,2*c*] and *E*[*Δ*_*b*_|*b*,*c*]. To be more precise, the following equation holds:

(4) $$\begin{array}{l} E[\Delta_{b}^{\prime }|b,c]=(1+q^{d+1})E[\Delta_{b}|b,2c]-q^{d+1}E[\Delta_{b}|b,c]. \end{array}$$

See Methods for the proof. When *c*→0, both *E*[*Δ*_*b*_|*b*,2*c*] and *E*[*Δ*_*b*_|*b*,*c*] approach *d*/2, which is the expected resolution when a deletion is detected with only one read pair. Therefore $E[\Delta_{b}^{\prime }|b,c]$ also approaches *d*/2 when *c*→0. On the other hand, when *c* approaches infinity, $E[\Delta_{b}^{\prime }|b,2c]$ approaches *E*[*Δ*_*b*_|*b*,2*c*] because *q*^*d* + 1^→0. In summary,

##### Theorem 2

$E[\Delta_{b}^{\prime }|b,c]$ is asymptotically equal to *E*[*Δ*_*b*_|*b*,2*c*] when *c*→0 or *c*→*∞*.

#### Trimming of deletion calls to improve resolution

**Figure 3:**
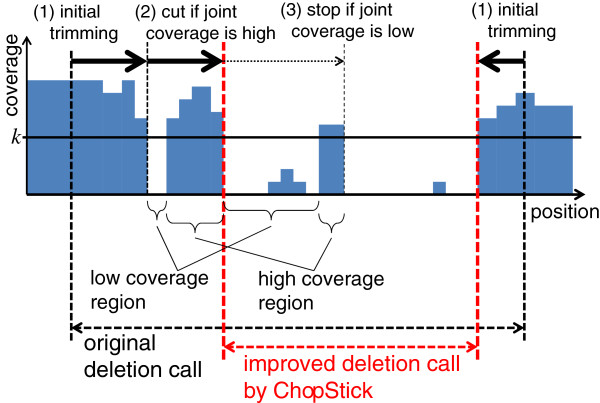
**Overview of trimming algorithm of ChopSticks.** Schematic illustration of the trimming algorithm of ChopSticks. ChopSticks trims ends of deletion calls that are not likely to be parts of deletions, according to their coverage. First, it trims high-coverage regions at the ends of deletion calls. Here, a *high-coverage region* is a region whose coverage is greater than a given parameter *k*. Second, it recognizes a high-coverage region separated by a low-coverage region and trims these regions if their joint coverage is deeper than *kf*, where *f* is another parameter. The second step is repeatedly conducted until the joint coverage becomes less than *kf* .

If all regions existing in the reference genome were covered by at least one read and there were absolutely no reads mapped to regions of homozygous deletions, the resolution of deletion calls could be quite easily improved by just trimming the ends of deletion calls that are covered by alignments of reads. Obviously, such a simple assumption does not hold in practical situations. First, coverage might be zero even in regions that actually exist in the genome, because no reads are obtained therein owing to the unevenness of the coverage or because reads cannot be uniquely mapped owing to repeat elements. Second, there might exist erroneous alignments in deleted regions because of incidental sequence similarity. Therefore, we developed the algorithm ChopSticks to carefully trim the ends of deletion calls (Figure 3). ChopSticks recognizes high-coverage regions close to the ends of deletion calls even if they are fragmented, and it repeatedly excludes the high-coverage regions from deletion calls. ChopSticks uses two parameters, *k* and *f*. The *k* parameter is a threshold used to distinguish high-coverage regions from low-coverage ones, and *f* determines the threshold of joint coverage of regions excluded from a deletion call. See Methods for details. Our implementation of ChopSticks is available on the Internet [19].

### Computational experiment

To evaluate the power of ChopSticks in improving the resolution of deletion calls, we conducted computational experiments. Let the *upstream difference* of a deletion call be *x*−*y*, where *x* is the position of the upstream end of the true deletion and *y* be that of the deletion call. Similarly, let the *downstream difference* of a deletion call be *y*^′^−*x*^′^, where *x*^′^ is the position of the downstream end of the true deletion and *y*^′^is that of the deletion call. By definition, the closer to zero a difference is, the better. A positive difference value indicates that the called breakpoint is outside the true deletion, whereas a negative value indicates that it is inside the true deletion. To evaluate ChopSticks, the results of ChopSticks have to be compared with the positions of true deletions. Therefore we need NGS reads of a genome whose SVs against the reference genome are known up to bp-level resolution. We conducted two experiments described below.

#### Simulated reads

In the first experiment, we evaluated ChopSticks with simulated NGS reads for which all SVs were known up to bp-level resolution. To obtain data as realistic as possible, we generated a genome sequence with SVs and simulated NGS sequences by using SV annotations published by Quinlan et al. [7]. The accession number of the SV annotations is [dbVar:nstd19]. First, we deleted regions of the reference genome sequence that were annotated as deletions by Quinlan et al. Next, we inserted random fragments whose number and distribution of lengths were the same as annotated deletions, assuming that deletions and insertions are symmetric. Then, we introduced single nucleotide substitutions into the simulated genome sequence and generated paired reads from it. We conducted this simulation and evaluation of ChopSticks for chromosome 1 of the reference mouse genome mm9. All paired reads were mapped to mm9 using Burrows-Wheeler aligner (BWA) [20]. Then we conducted SV analysis by using SV detection tools from each of categories described in the Background section: BreakDancer [5] of threshold-based RP methods, MoDIL [8] of distribution-based RP methods, CLEVER [9] of graph-based RP methods, CNVnator [11] of RD methods, and Pindel [12] of SR methods. After that, we applied ChopSticks to their results.

**Figure 4:**
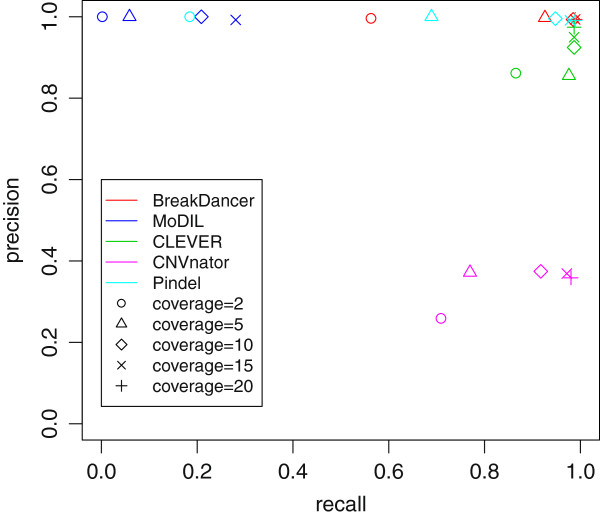
**Recall and precision of results of SV detection tools.** BreakDancer and CLEVER achieved relatively good recall for all coverage, while recall of MoDIL was low. Although recall of CNVnator was not bad, its precision was low. The recall of an SR method Pindel was good when coverage was high, but it was insufficient when coverage was low.

**Figure 5:**
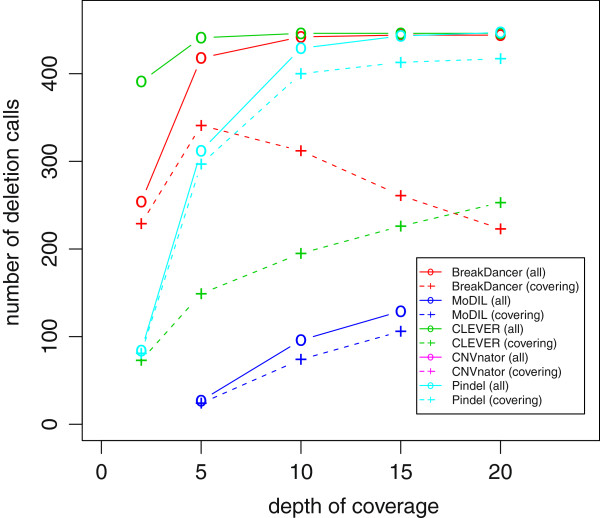
**Number of deletion calls covering the whole of true deletions.** Solid lines and circles show the number of all deletion calls generated by each tool, whereas dashed lines and ‘+’ symbol s show the number of deletion calls covering the whole of true deletions. Most of the deletion calls of MoDIL, CNVnator (expanded by the window size), and Pindel covered the whole of true deletions. On the other hand, many CLEVER results did not always contain the whole of true deletions, while median of the distribution of predicted breakpoints was close to the true breakpoints as shown in Figure 10. BreakDancer results for high coverage data did not always contain true deletions either. Predicted breakpoints of BreakDancer approached true breakpoints as the depth of coverage increases, and sometimes intruded into true deletions when coverage was high.

Before applying ChopSticks, we examined the ability of SV detection tools to detect 460 deletions in chromosome 1 of the simulated mouse genome. We say that a deletion call is *correct* if it overlaps exactly one true deletion while the true deletion in turn overlaps exactly one deletion call. We show the number of called and correct SV calls in Table 1. We also show their *recall* (the number of correct deletion calls divided by the number of true deletions) and *precision* (the number of correct deletion calls divided by the number of all deletion calls) in Figure 4. The recall of BreakDancer and CLEVER was relatively good for all of tried coverage values, whereas the recall of Pindel was satisfactory only when coverage was high. The recall of MoDIL was low for all coverage values tried. Although almost all deletions called by these methods were correct, CNVnator generated numerous false positives (Table 1). Because ChopSticks is developed to correct breakpoints outside true deletions, we counted the number of deletion calls that cover the whole of true deletions. As shown in Figure 5, most of the deletion calls by MoDIL, CNVnator, and Pindel covered the whole of true deletions. However, a significant portion of BreakDancer and CLEVER results did not cover the whole of true deletions. Note that ChopSticks is harmless to these deletion calls because ChopSticks does not trim them when there are no alignments in true deletions.

**Table 1:** Results of SV detection obtained by BreakDancer, MoDIL, CLEVER, CNVnator, and Pindel

	**Depth of coverage**				
**SV caller**	**2**	**5**	**10**	**15**	**20**
BreakDancer	259/260	426/427	453/456	455/458	455/458
MoDIL	1/1	27/27	96/96	129/130	–/–
CLEVER	398/462	449/525	454/491	454/478	454/466
CNVnator	326/1,258	354/952	422/1,127	447/1,211	451/1,258
Pindel	85/85	317/317	436/438	450/454	456/456

Results of deletion calls by BreakDancer, MoDIL, CLEVER, CNVnator, and Pindel. The values to the left of ”/” are the numbers of *correct* deletion calls, where a *correct* deletion call is the one that overlaps with exactly one true deletion, which, in turn, only overlaps with the deletion call; the values to the right of ”/” are the numbers of all deletion calls. BreakDancer and CLEVER results were good in both sensitivity and specificity. CNVnator generated numerous false positives, while Pindel suffered from low coverage. MoDIL missed lots of deletions.

Next, we applied ChopSticks to the results of SV detection tools. After that, we examined how well the resolution of deletion calls was improved. We tested ChopSticks for *k*=1,2,…,5 and *f*=0.1,0.2,…,1.0. We evaluated differences at both the upstream and downstream ends of deletions, and found that the results were similar. Therefore we only present the results at upstream ends.

##### Resolution improvements for BreakDancer deletion calls

**Figure 6:**
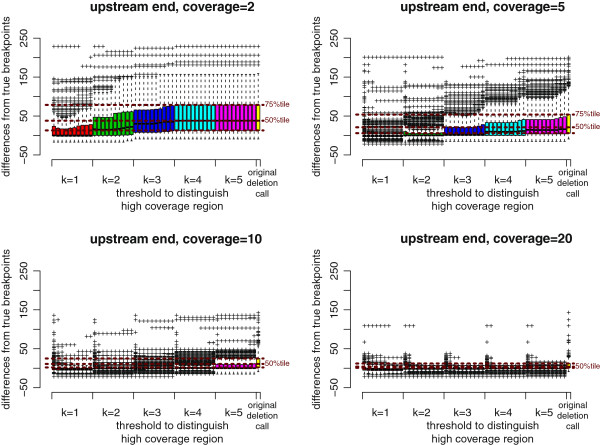
**BreakDancer results improved by ChopSticks.** Box-and-whisker plots of upstream differences of deletion calls obtained by BreakDancer and those improved by ChopSticks. The red, green, blue, light blue, and magenta boxes correspond to *k* values of 1, 2, 3, 4, and 5, respectively, and the rightmost yellow box corresponds to the original results of BreakDancer. Among boxes of the same color, from left to right, *f*=0.1, 0.2, …, 1.0. Brown horizontal dashed lines indicate the values of 25%, 50%, and 75% tiles of differences of original deletion calls from below to above, respectively. The results in this figure indicate that ChopSticks clearly improved the resolution of the original BreakDancer results. When the coverage was low, small *k* values were effective in improving the resolution. When coverage was high, the resolution was also improved for large *k* values. Therefore, when the coverage is high, we recommend using large *k* values to avoid erroneous alignments of NGS reads and the genome. We omitted the results for coverage=15 because they were similar to those for coverage=20.

**Figure 7:**
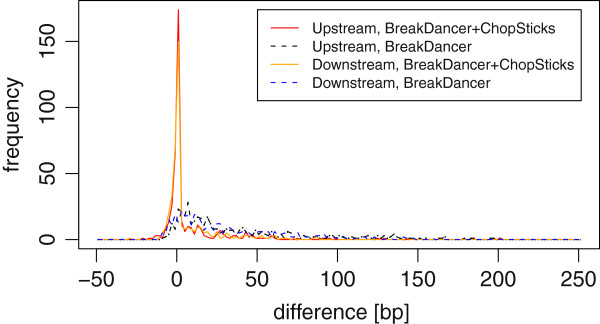
**Distribution of differences of BreakDancer results and those improved by ChopSticks.** The distribution of differences of ChopSticks results concentrated around zero, whereas that of BreakDancer results had long tail in 0–50 bp. Here, *k*=2, *f*=0.5, and coverage=5. Each frequency corresponds to the number of differences in bins of 2 bp.

**Figure 8:**
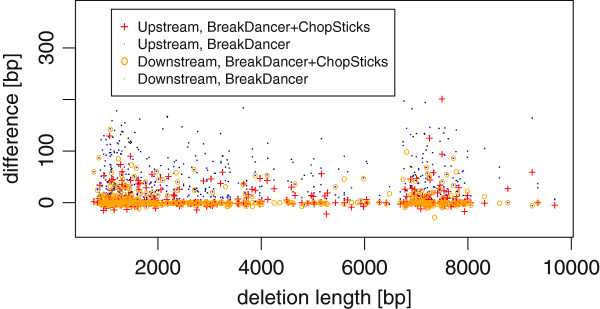
**Scatter plot of deletion lengths and differences of deletion calls.** No correlation between deletion lengths and differences was observed (*r*^2^=0.056). ChopSticks worked well regardless of deletion lengths. Here, *k*=2, *f*=0.5, and coverage=5.

As shown in Figure 6, the resolution of deletion calls was clearly improved by using ChopSticks. The original BreakDancer results was successfully corrected, which is also clear in Figure 7. When coverage was low, the resolution was well improved for small *k* values. When coverage was high, the resolution was also improved for large *k* values. Therefore, when the coverage is high, we recommend using large *k* values to ignore erroneous alignments. As shown in Figure 8, ChopSticks worked well regardless of deletion lengths.

##### Resolution improvements for MoDIL deletion calls

**Figure 9:**
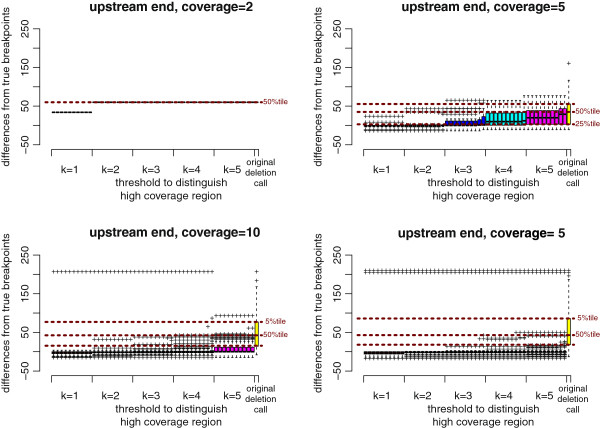
**MoDIL results improved by ChopSticks.** Box-and-whisker plots of upstream differences of deletion calls obtained by MoDIL and those improved by ChopSticks. The format of this plot is exactly the same as that in Figure 6, except that results for coverage=15 were shown instead of those for coverage=20. The results in this figure indicate that ChopSticks can also improve the resolution of MoDIL results.

As shown in Figure 9, the resolution of deletion calls by MoDIL was also improved by using ChopSticks. We omitted evaluation of MoDIL for coverage=20 because MoDIL was very slow (See Methods).

##### Resolution improvements for CLEVER deletion calls

**Figure 10:**
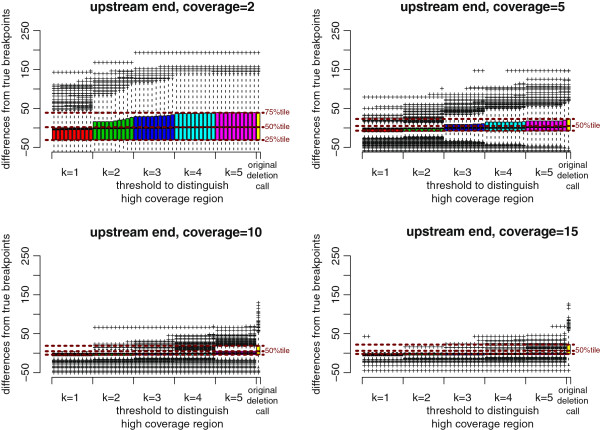
**CLEVER results improved by ChopSticks.** Box-and-whisker plots of upstream differences of deletion calls obtained by CLEVER and those improved by ChopSticks. The differences were successfully corrected. Note that a significant portion of breakpoints predicted by CLEVER were inside the true deletion. Nonetheless, ChopSticks selectively trimmed predicted breakpoints outside true deletions, and left those inside untouched.

**Figure 11:**
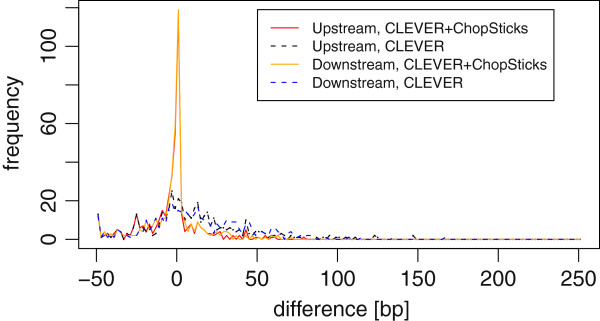
**Distribution of differences of CLEVER results and those improved by ChopSticks.** The distribution of differences of CLEVER results had long tail in 0–50 bp, whereas that improved by ChopSticks concentrates around zero. Here, *k*=2, *f*=0.5, and coverage=5. Each frequency corresponds to the number of displacements in bins of 2 bp.

The resolution of deletion calls by CLEVER was also improved by using ChopSticks. As mentioned above, deletion calls of CLEVER do not always cover the whole of true deletions. Nonetheless, as shown in Figure 10 and 11, ChopSticks successfully improved resolution of CLEVER results by selectively correcting predicted breakpoints outside true deletions.

##### Resolution improvements for CNVnator deletion calls

**Figure 12:**
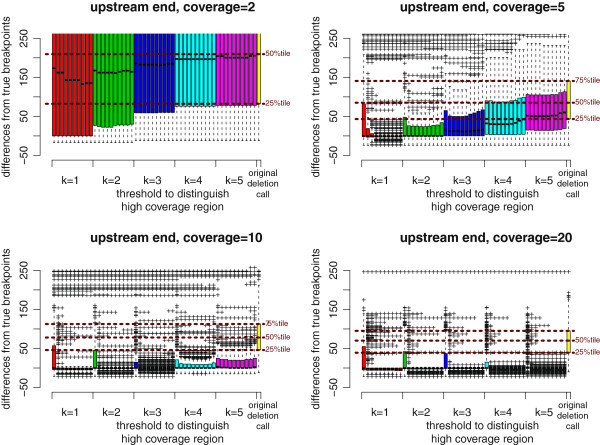
**CNVnator results improved by ChopSticks.** Box-and-whisker plots of upstream differences of deletion calls obtained by CNVnator and those improved by ChopSticks. The format of this plot is exactly the same as that in Figure 6. We expanded the original deletion calls of CNVnator outward by the window size (50 bp) because ChopSticks assumes that predicted breakpoints are outside true deletions. The results in this figure indicate that ChopSticks can improve the resolution of CNVnator results if predicted positions of breakpoints are within a few hundreds of bases from true breakpoints.

Because RD methods call SVs by examining coverages in windows of a fixed size, the positions of breakpoints predicted by the RD methods have unavoidable ambiguity and they might be either inside or outside true deletions. Because ChopSticks assumes that predicted breakpoints are outside true deletions, we applied ChopSticks after we expanded deletion calls of CNVnator at both ends by the window size. As shown in Figure 12, the results of CNVnator were successfully improved. This result indicates that ChopSticks is also available for RD methods in addition to RP methods.

##### Results of ChopSticks applied to Pindel deletion calls

**Figure 13:**
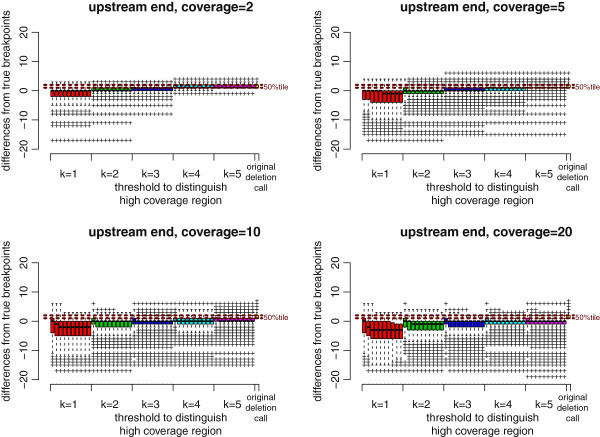
**Pindel results and those modified by ChopSticks.** Box-and-whisker plots of upstream differences of deletion calls obtained by Pindel and those modified by ChopSticks. The format of this plot is exactly the same as in Figure 6. The results in this figure indicate that ChopSticks should not be applied to the Pindel results because the resolution of the Pindel results is already quite high.

Owing to the SR signature that allows Pindel to detect SVs at bp-level resolution, the positions of breakpoints obtained with Pindel were quite accurate. When ChopSticks was applied to the results of Pindel, the results became slightly worse than the original Pindel results, as shown in Figure 13, although differences remained close to zero in most cases. Note that the recall of Pindel was not satisfactory when coverage is low, as shown in Figure 4. ChopSticks is useful in cases where deletions missed by Pindel are analyzed.

#### Real Illumina reads of DBA/2J

In the second experiment, we evaluated ChopSticks using the real NGS sequences of Quinlan et al. [7]. The sample was taken from a female mouse of the DBA/2J strain, whose genome contains SVs against the reference genome of the C57BL/6J strain [21]. The read sequences were available from the NCBI Sequence Read Archive (SRA) database [22]. The accession number of the read sequences is [SRA:SRA010027]. To evaluate the results of ChopSticks, we need bp-level SV annotations of DBA/2J as well. Therefore we generated deletion calls at bp-level resolution using Sanger reads in a manner similar to that of Quinlan et al. See Methods for details. Our deletion calls are available at the dbVar database under accession no. [dbVar:nstd70].

We tried the five SV detection tools used in the previous experiment, and found that MoDIL, CNVnator and Pindel missed the most of deletions detected with Sanger reads. These methods seemed to suffer from the low depth of coverage and short read lengths. Therefore, we hereafter only describe results of ChopSticks applied to BreakDancer and CLEVER results.

##### Resolution improvements for BreakDancer deletion calls

**Figure 14:**
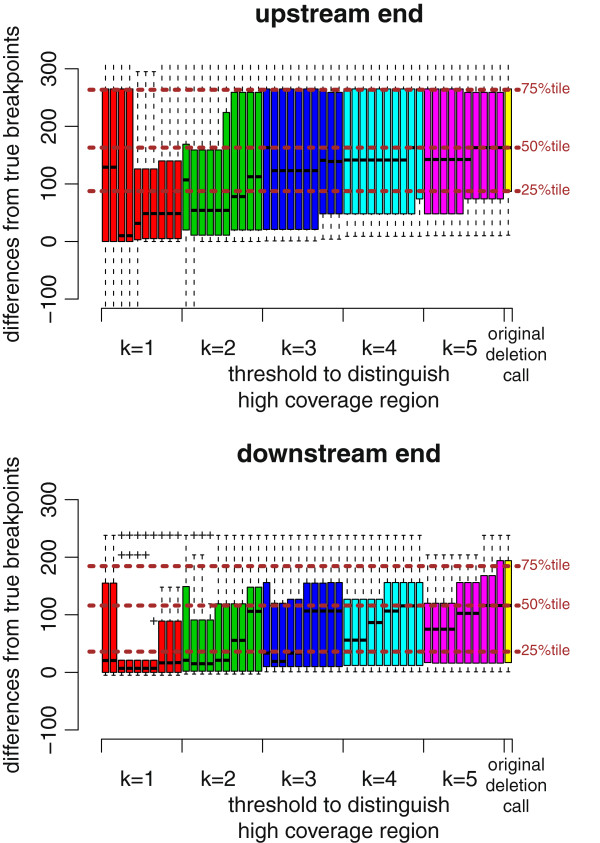
**BreakDancer results for DBA/2J reads improved by ChopSticks.** Box-and-whisker plots of upstream and downstream differences of deletion calls obtained by BreakDancer and those improved by ChopSticks. The results in this figure indicate that ChopSticks can improve the resolution of deletion calls for real sequences. Although ChopSticks trimmed upstream ends of a few deletion calls too much when *k*=1 or *k*=2 and *f* was small, such problems quickly disappeared for greater *k* and *f* values.

**Figure 15:**
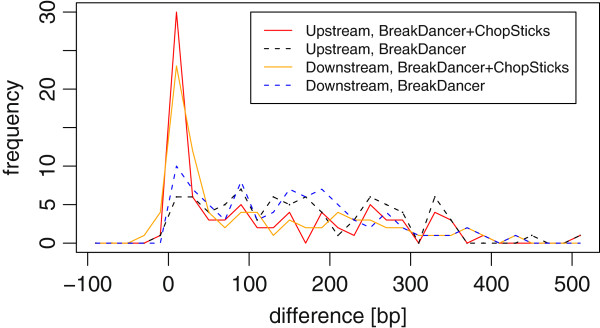
**Distribution of differences of BreakDancer results and those improved by ChopSticks.** The distribution of differences of BreakDancer results had long tail in 0–400 bp, whereas that improved by ChopSticks concentrates around zero and frequencies in the long tail were reduced. Here, *k*=2, *f*=0.5. Each frequency corresponds to the number of differences in bins of 20 bp.

Figure 14 shows the differences between BreakDancer results and those improved by using ChopSticks. As the previous experiment where simulated NGS reads were used, the differences obtained with real NGS reads were reduced. The median and differences less than the median clearly shifted toward zero, which is also clear in Figure 15. Although ChopSticks trimmed some deletion calls into those based on Sanger reads when *k*=1 or *k*=2 and *f* was small, this problem quickly disappeared as *k* or *f* became larger. No correlation between deletion lengths and the performance of ChopSticks were observed (*r*^2^=0.021). Although we generated 525 deletion calls by using Sanger reads, only 83 of them were found by BreakDancer. There were at least two reasons for this difference in numbers. First, it is difficult to find small deletions because read pairs spanning small deletions might not be recognized as discordant pairs. Second, a lot of deletion calls based on Sanger reads had fewer than two NGS-read pairs spanning them. Such deletion calls would be missed because BreakDancer deletion calls must be supported by at least two pairs when the default parameters are used, in order to reduce false positives. For this data set, 82 of all 83 deletion calls generated by BreakDancer contained the whole of deletions predicted with Sanger reads.

##### Resolution improvements for CLEVER deletion calls

**Figure 16:**
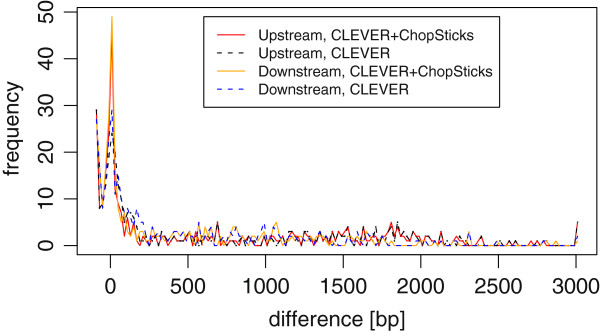
**Distribution of differences of CLEVER results and those improved by ChopSticks.** ChopSticks corrected some of breakpoints predicted by CLEVER so that the peak at zero became stronger. However, the distribution of differences of CLEVER results had long tail in 0–3000 bp and it was difficult for ChopSticks to correct such large differences. Here, *k*=2, *f*=0.5. Each frequency corresponds to the number of differences in bins of 20 bp.

CLEVER detected much more (347) deletions than BreakDancer. The results of CLEVER were also improved by ChopSticks as shown in Figure 16, where the peak around zero became stronger. However, it was difficult for ChopSticks to correct positions of breakpoints when they were away from those predicted with Sanger reads by hundreds of bases.

## Conclusions

We have presented a new method called ChopSticks to improve the resolution of predicted positions of deletions. The key idea is to exploit both concordant read pairs and discordant ones. According to our theoretical analysis, the resolution of our method is quite similar to that of threshold-based RP methods but with double coverage. In an experiment on simulated NGS reads, ChopSticks clearly improved the results of BreakDancer, MoDIL, CLEVER, and CNVnator. Although the resolution of Pindel results is quite high, ChopSticks works well even for low-coverage data where recall of Pindel is not sufficient. The effectiveness of ChopSticks was also confirmed by performing an experiment on real Illumina reads. Despite a number of methods proposed for detecting SVs [2-4], there is no one-stop method that simultaneously achieves high sensitivity, high specificity, high resolution, and robustness for low-coverage data. Therefore a combination of SV detection methods is required, and ChopSticks can play an important role because it uses new independent information ignored in other methods.

As a future work, we consider to develop a method to distinguish homozygous deletions from heterozygous ones and to apply ChopSticks to the former. With this approach, ChopSticks will be available for more applications.

## Methods

### Derivation of theoretical estimation of resolution

Because the resolution at downstream ends of deletions can be estimated symmetrically, we only analyze the resolution at upstream ends. Let *P*_*b*_be the probability that a breakpoint *b* is successfully included in a deletion call by a threshold-based RP method. If *b* is detected, there exists an upstream discordant read within *d* bases from *b*. Therefore,

$$P_{b}=1-q^{d+1}.$$

We derive the expected distance between the true ends of deletions and the predicted ones in a manner similar to Bashir’s analysis [23]. For 0≤*j*≤*d*, Bashir et al. defined *A*_*j*_as an event in which *b* is detected and an upstream read of a discordant pair is exactly *j* bases upstream of *b*. The probability that *A*_*j*_occurs is

$$\begin{array}{l} \text{Pr}(A_{j})=(1-q)q^{j}. \end{array}$$

Consequently,

$$\begin{array}{l} E[\Delta_{b}|b,c]\\ =\frac{1}{P_{b}}\underset{0\leq j\leq d}{\sum }j\text{Pr}(A_{j})\\ =\frac{1-q}{1-q^{d+1}}S(q,d). \end{array}$$

Similarly, we define $A_{j}^{\prime }$ as an event wherein *b* is detected and the closest read upstream of *b* is exactly *j* bases apart. There are two mutually exclusive cases: (i) at least one of the closest reads is an upstream discordant read or (ii) all the closest reads are concordant reads. In the latter case, we have to consider the joint probability of the following events. 

• A concordant read exists at *j* bases upstream of *b*, the probability of which is 1−*q*.

• No read nearer than the closest concordant read exists, the probability of which is *q*^2*j*^.

• No discordant read exists at *j* bases upstream of *b*, the probability of which is *q*.

• There must exist an upstream read of discordant pairs whose alignment ends in a region that is *j* + 1 to *d* bases upstream of *b* so that *b* is successfully included in a deletion call, the probability of which is 1−*q*^*d*−*j*^.

Therefore,

$$\begin{array}{ll} \text{Pr}(\overset{\prime }{\underset{j}{A}})=(1-q)q^{2j}+(1-q)q^{2j}q(1-q^{d-j}) & \\ \;=(1-q^{2})q^{2j}-q^{d+1}(1-q)q^{j}. \end{array}$$

Consequently,

$$\begin{array}{l} E[\Delta_{b}^{\prime }|b,c]=\frac{1}{P_{b}}\underset{0\leq j\leq d}{\sum }j\text{Pr}(\overset{\prime }{\underset{j}{A}})\\ \;=\frac{1}{1-q^{d+1}}\left(\;(1-q^{2})S(q^{2},d)\right)\\ \;\left(\;-q^{d+1}(1-q)S(q,d)\right). \end{array}$$

#### Proof of Theorem 1

From Equation (1), *E*[*Δ*_*b*_|*b*,2*c*] can be obtained by replacing *q*with *q*^2^ in Equation (2):

(5) $$\begin{array}{l} E[\Delta_{b}|b,2c]=\frac{1-q^{2}}{1-q^{2(d+1)}}S(q^{2},d). \end{array}$$

From Equations (2), (3), and (5), Equation (4) can be obtained.

#### Proof of Theorem 2

First, we consider a case where *c*→0. Because *q*→1 by Equation (1),

$$S(q,d)\to \overset{d}{\underset{j=0}{\sum }}j=\frac{d(d+1)}{2}.$$

Besides,

$$\frac{1-q}{1-q^{d+1}}=\frac{1}{1+q+q^{2}+\cdots +q^{d}}\to \frac{1}{d+1}.$$

Therefore, all of $E[\Delta_{b}^{\prime }|b,c]$, *E*[*Δ*_*b*_|*b*,2*c*], and *E*[*Δ*_*b*_|*b*,*c*] approach *d*/2 by Equation (4). On the other hand, when *c*→*∞*, *q*^*d* + 1^approaches 0. In consequence, the right hand side of Equation (4) approaches *E*[*Δ*_*b*_|*b*,2*c*] when *c*→0 or *c*→*∞*.

### Mapping to the genome

We mapped paired reads to the mm9 reference genome sequences of *Mus musculus* using BWA version 0.5.9 [20] with default parameters. The target genome sequences involved in our experiment included all chromosomes of mm9 except chromosome Y, assuming cases where a female mouse was analyzed [7,21].

#### Simulated NGS sequences

To focus on uniquely mapped reads for BreakDancer, MoDIL, CLEVER, and ChopSticks, we removed paired reads if the mapping quality (MAPQ) score was zero for at least one of the two reads of a pair. For CNVnator and Pindel, we used the result of BWA without filtering. We show the total length of reads and the number of aligned reads in Table 2.

**Table 2:** Number of bases and number of reads of simulated data set

	**Depth of coverage**				
	**2**	**5**	**10**	**15**	**20**
Total number of bases	394,391,200	985,978,000	1,971,956,000	2,957,934,000	3,943,912,000
Number of reads	3,943,912	9,859,780	19,719,560	29,579,340	39,439,120
Number of mapped reads	3,677,398	9,194,942	18,391,288	27,587,970	36,783,348

Summarized statistics of simulated NGS reads and their alignments to mm9. On the third row, we only counted read pairs whose reads were both mapped uniquely.

#### Real DBA/2J sequences

We split the data set of NGS reads into 275 subsets, and mapped each of them with an independent BWA process and merged the results. Then we removed reads whose MAPQ score was zero for at least one of the two reads of a pair. We show the total length of reads and the number of aligned reads in Table 3.

**Table 3:** Number of bases and number of reads of DBA/2J data set

Total number of bases	13,050,980,662
Number of reads	330,462,408
Reads of uniquely mapped pairs	149,021,716
Reads of uniquely mapped pairs (chromosome 1)	10,316,525

Summarized statistics of NGS reads of the DBA/2J strain [7] and their alignments to mm9.

### Trimming algorithm of ChopSticks

**Figure 17:**
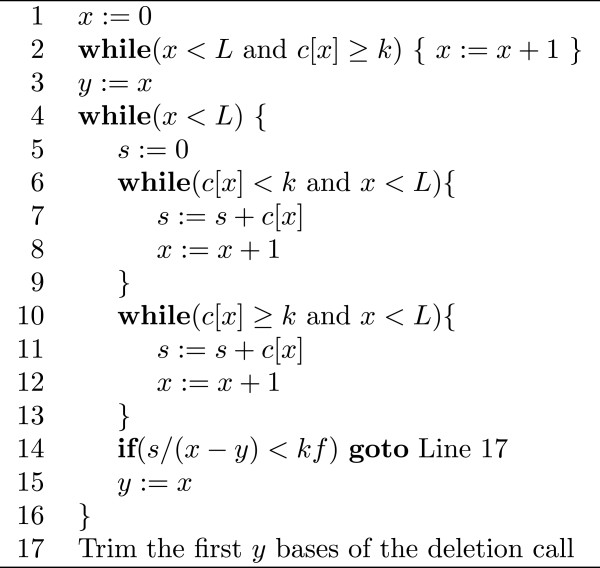
**Pseudocode of trimming algorithm.** Pseudocode of the trimming algorithm of ChopSticks. Here, *L* is the length of the deletion call being processed, *k* is a threshold used to discriminate high-coverage regions from low-coverage ones, and *f* is a parameter that determines the threshold of the coverage of regions to be trimmed. The variable *x* represents the position of the base being examined, and the variable *y* represents the length of a region to be trimmed. The value *c*[*x*] is the coverage at the *x*-th base in the deletion call, while *s* keeps the sum of *c*[*x*] values.

The coverage outside a deletion should be higher than that inside it. Therefore ChopSticks repeatedly recognizes a high-coverage region in a deletion call that is likely a continuation of a high-coverage region outside the deletion. We show in Figure 17 the trimming algorithm executed by ChopSticks for upstream ends. Here is a brief description of the algorithm: **Line 2:** Skip a high-coverage region at the end of the deletion call. **Lines 6–9:** Go through a low-coverage region. **Lines 10–13:** Go through a high-coverage region. **Line 14:** If the joint coverage is low, exit the loop. **Line 17:** Trim regions which the algorithm has gone through.Trimming of the downstream ends is conducted symmetrically.

### Data for computational experiments

To evaluate our method, we need NGS sequences and reliable bp-level positions of breakpoints. There were six SV studies of inbred mice (nstd5, 7, 15, 18, 19, and 48) in the dbVar database [22] when we accessed it on April 1, 2012. However, none of them provides accurate bp-level positions of breakpoints. Therefore, we evaluated ChopSticks using the following two data sets.

#### Simulated NGS reads

**Figure 18:**
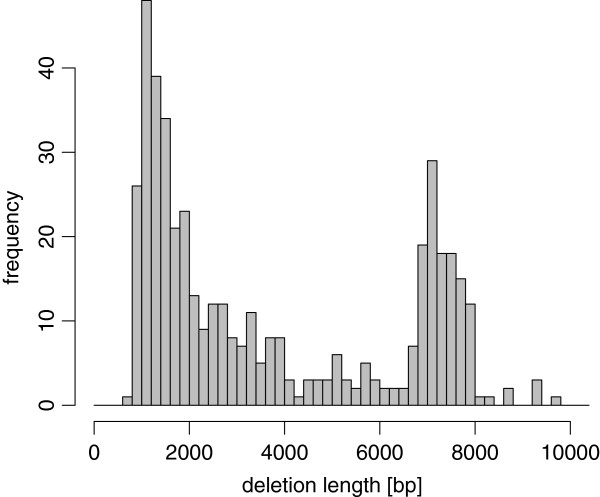
Distribution of deletion lengths in our simulation.

We artificially introduced deletions and insertions into the mm9 reference genome and then generated simulated NGS reads using the modified genome. To obtain most realistic simulated sequences, we built a simulated genome sequence using SV annotations generated by Quinlan et al. [7], which are available from the dbVar database under accession no. [dbVar:nstd19]. First, we deleted regions annotated as deletions in [dbVar:nstd19] from the mm9 reference genome sequence of chromosome 1. We show the distribution of lengths of deletions in Figure 18. Second, we inserted fragments consisting of randomly chosen bases so that the number and the distribution of lengths of inserted fragments were the same as those of deletions, assuming that the genome to be analyzed and the reference genome are affected symmetrically by deletions and insertions. Third, we introduced random single nucleotide substitutions with a probability of 1.0×10^−4^ at each base. Finally, we generated paired reads from the modified genome sequence so that the read length was 100 bp and the average and the standard deviation of distances of paired reads were 200 bp and 50 bp, respectively. We generated five sets of simulated NGS reads whose depth of coverage were 2, 5, 10, 15, and 20, respectively.

#### NGS reads of Quinlan et al. and deletion calls based on Sanger reads

**Figure 19:**
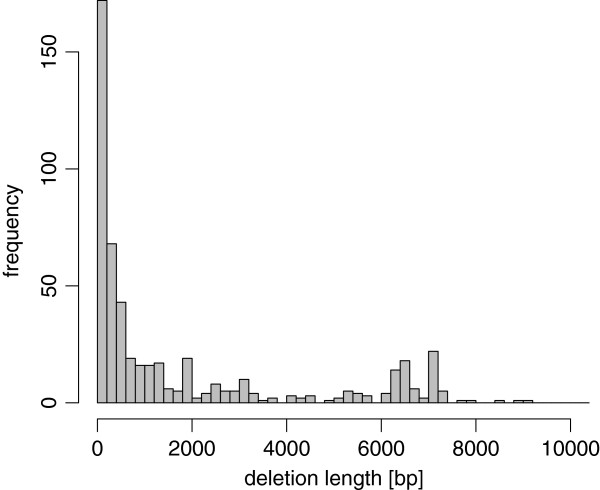
Distribution of deletion lengths detected with Sanger reads.

We generated our own bp-level deletion calls by using publicly available Sanger reads of the DBA/2J strain. From the NCBI trace archive, we retrieved all 7,998,826 Sanger reads of whole-genome shotgun sequencing for the DBA/2J strain. We mapped these Sanger reads to chromosome 1 of mm9 by MegaBLAST [24], and we searched for Sanger reads that were split into two parts and aligned uniquely on the same strand and in the right order. There were 763 reads that indicated deletions whose lengths were at least 50 bp. By merging redundant ones, we obtained 525 deletion calls. These deletion calls are available in the dbVar database under accession no. [dbVar:nstd70]. We show the distribution of their lengths in Figure 19. NGS sequences of the DBA/2J strain generated by Quinlan et al. are available in the SRA database [22] under accession no. [SRA:SRA010027].

### Parameters for SV detection tools and evaluation of their results

We executed BreakDancer with default parameters, and Pindel with an expected template size of 432 bp because the median fragment size was 432 bp according to Quinlan et al. [7]. For CNVnator, we tested three window sizes: 50 bp, 100 bp, and 200 bp. Because the recall of window size 50 bp outperformed those of window sizes 100 bp and 200 bp for our simulated data when coverage was 2, we used results of window size 50 bp for evaluation. Because CLEVER tends to generate deletion calls duplicatedly with slightly different positions, we chose the best one for those overlapping with true deletions in order to estimate the upper limit of the accuracy of CLEVER. We divided the chromosome 1 of mm9 into 5.1 Mbp fragments in a manner such that franking fragments share 0.1Mbp, and applied MoDIL to each fragments, because MoDIL was quite slow as reported previously [9]. We omitted evaluation of MoDIL for coverage=20.

To compare the positions of true and predicted deletions, we used BEDTools [25].

## Competing interests

The authors declare that they have no competing interests.

## Authors’ contributions

TY conceived the project, invented and implemented the algorithms, and performed the computational analysis. SS assisted TY in conducting experiments. MN and SM critically revised the manuscript. All authors read and approved the final manuscript
